# Development and validation of a prokaryotically expressed foot-and-mouth disease virus non-structural protein 2C'3AB-based immunochromatographic strip to differentiate between infected and vaccinated animals

**DOI:** 10.1186/1743-422X-8-186

**Published:** 2011-04-23

**Authors:** Lei Wu, Tao Jiang, Zeng-Jun Lu, Ya-Min Yang, Pu Sun, Zhong Liang, Dong Li, Yuan-Fang Fu, Yi-Mei Cao, Xiang-Tao Liu, Zai-Xin Liu

**Affiliations:** 1 State Key Laboratory of Veterinary Etiologic Biology, National Foot-and-Mouth Disease Reference Laboratory of China, Key laboratory of Animal Virology of Ministry of Agriculture, Lanzhou Veterinary Research Institute, Chinese Academy of Agricultural Sciences, Lanzhou, 730046, Gansu, China

**Keywords:** an immunochromatographic strip, foot-and-mouth disease virus, colloidal gold-labeled 2C'3AB antigen, prokaryotic expression, development, validation

## Abstract

**Background:**

Foot-and-mouth disease (FMD) is an extremely contagious viral disease of cattle, pigs, sheep, goats, and many cloven-hoofed wild animals. FMDV serotypes O and Asia 1 have circulated separately in China during the last fifty years, and eliminating infected animals and vaccination are the main policies to prevent and control FMD. Antibodies to NSPs exist in infected animals, and were utilized to differentiate between infected and vaccinated animals. The reliability of detection of 3AB or 3ABC antibodies is higher than that of other NSPs. The test of 3AB is still credible because 3C protein's immunogenicity is the weakest. The 2C protein, immediately N-terminal of 3AB, was used to differentiate between infected and vaccinated animals. The use of the immunochromatographic strip is facile for clinical laboratories lacking specialized equipment and for rapid field diagnosis.

**Results:**

In this study, an immunochromatographic strip with non-structural protein (NSP) 2C'3AB was developed and validated to differentiate foot-and-mouth disease infected from vaccinated animals. A part of N-terminal of 2C protein gene and whole 3AB gene were connected and prokaryotically expressed as the antigens labeled with colloidal gold was used as the detector, the 2C'3AB protein and rabbits anti-2C'3AB antibodies were blotted on the nitrocellulose(NC) membrane for the test and control lines, respectively. 387 serum samples were collected to evaluate the characteristics of the strip in comparison with existing commercial 3ABC antibody ELISA kit. The coincidence rate of pigs negative serum, pigs vaccinated serum, pigs infected serum was 100%, 97.2%, 95.0%, respectively. The coincidence rate of cattle negative serum, cattle vaccinated serum, cattle infected serum was 100%, 96.7%, 98.0%, respectively. The **c**oincidence rate of sheep negative serum, sheep infected **s**erum was 97.6%, 96.3%, respectively. The strip was shown to be of high specificity and sensitivity, good repeatability and stability.

**Conclusion:**

These data suggest that the immunochromatographic strip is a useful tool for rapid on-site diagnosing animals infected foot-and-mouth disease virus.

## Introduction

Foot-and-mouth disease (FMD) is an extremely contagious viral disease of cattle, pigs, sheep, goats, and many cloven-hoofed wild animals. The disease is widespread and causes significant economic losses. The causal agent, FMD virus (FMDV), is a positive-sense single-stranded RNA virus that is a member of the genus Aphthovirus in the family Picornaviridae and occurs as seven distinct serotypes throughout the world: A, O, C, Asia1 and South African Territories (SAT) 1-3. The non-structural proteins (NSPs) are conserved amongst the seven serotypes[1,2]. FMDV serotypes O, Asia 1 and A have circulated separately in China during the last fifty years[3-6], and eliminating infected animals and vaccination are the main policies to prevent and control FMD. Antibodies to NSPs exist in infected animals, and were utilized to differentiate between infected and vaccinated animals.

The reliability of detection of 3AB or 3ABC antibodies is higher than that of other NSPs[7]. The test of 3AB is still credible because 3C protein's immunogenicity is the weakest[8]. The 2C protein, immediately N-terminal of 3AB, was used to differentiate between infected and vaccinated animals[9]. The 3ABC indirect ELISA kit was developed and commercialized in our laboratory[10,11]. The use of the immunochromatographic strip is facile for clinical laboratories lacking specialized equipment and for rapid field diagnosis[12].

## Materials and methods

### Serum samples

54 cattle positive sera, 36 cattle negative sera, 94 multi-vaccinated sera; 20 swine positive sera, 51 swine negative sera, 36 multi-vaccinated sera; 54 ovine positive sera, 42 ovine negative sera. Reference positive and negative sera were provided by national foot-and-mouth disease reference laboratory of China.

### Main reagents

H_4_AuCl_4_·4H_2_O and BSA were purchased from Sigma, NC membrane and glass fiber paper were purchased from Schleicher&Schuell.

### The expression and purification of 2C'3AB recombination protein

**Figure 1:**
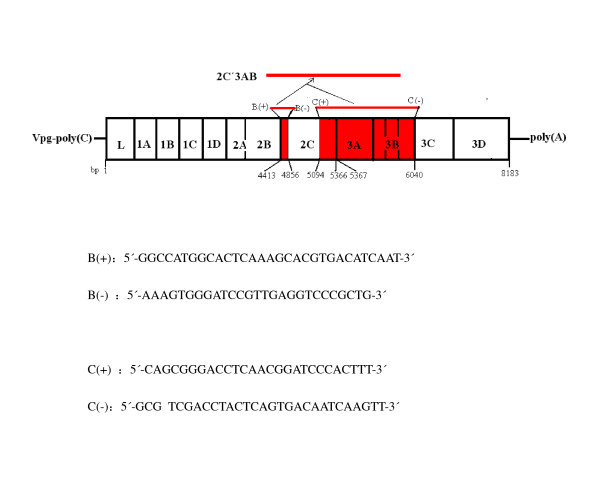
**The sequence and position of primers used for PCR amplification of sequences encoding the N-terminal region of 2C and the 3AB proteins**.

**Figure 2:**
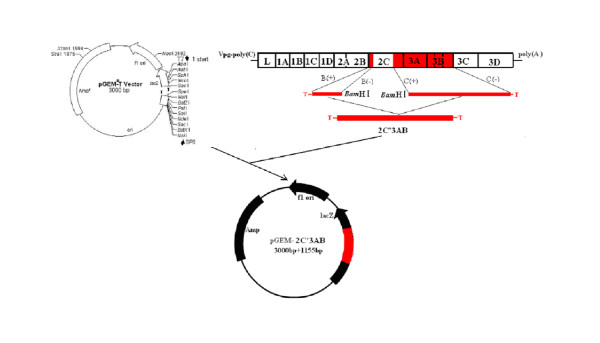
**The schematic construction of recombinant plasmid pGEM-2C'3AB**.

**Figure 3:**
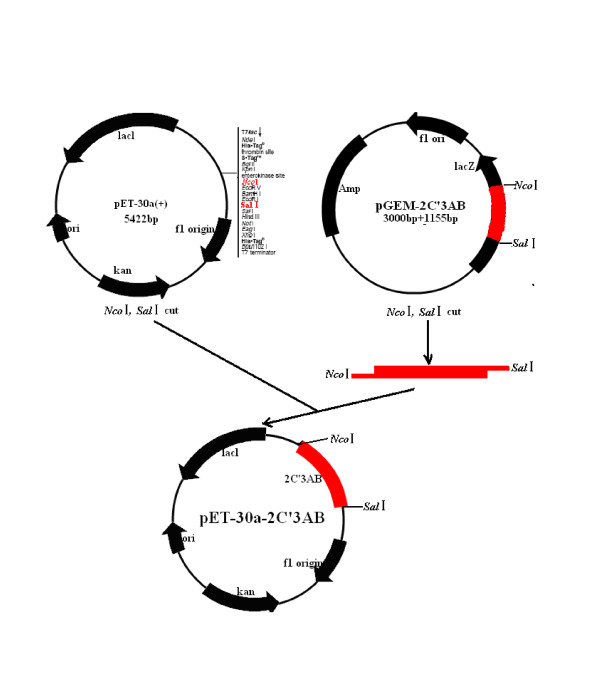
**The schematic construction of recombinant expression plasmid pET-30a-2C'3AB**.

Two couples of primers(see Figure 1) were designed with DNAstar and Oliogo6.0 softwares based on the sequence of FMDV O/China/99(Genbank No.AF506822) to amplify 2C'3AB gene using standard protocols. The 2C'3AB PCR product was ligated into pGEM-T (pGEM-2C'3AB; see Figure 2). pGEM-2C'3AB was restricted with NcoI and SalI, the insert gel purified and ligated into pET-30a, similarly restricted, to form pET-30a-2C'3AB (Figure 3). E. coli strain BL21(DE3)pLysS was transformed with pET-30a-2C'3AB. Isopropylthiogalactoside (IPTG) was added to induce expression of the 2C'3AB protein. The soluble 2C'3AB polypeptide in the lysate was purified by a HisTrap HP affinity chromatography column. SDS-PAGE and Western blotting were used to detect the expression product[1]. The concentration of the purified 2C'3AB protein was measured with BCA (Bicinchoninic Acid) protein quantitative kit (Beyotime, Shanghai)

### Preparation and identification of 2C'3AB antibody

Six rabbits were separated into three groups (two rabbits/group), vaccinated hypodermically four times (14 days interval each time) with the same dose(350ug/rabbit) of 2C'3AB antigen but different adjuvants(See Table 1). Rabbit sera were collected seven days after fourth vaccination. The sera were doubly diluted serially from 1:200 to detect their titers with indirect ELISA, The serum is positive when *OD*_*450nm(sample)*_*/OD*_*450nm(negative control)*_≥2.1, and its titer is the highest dilution. The 2C'3AB IgG was purified by ammonium sulfate precipitation method, and identified with SDS-PAGE and Western blot.

**Table 1:** Immunization protocol of rabbits

Immunization times	Group A (No.1, No.2)		Group B (No.3, No.4)		Group C (No.5, No.6)	
	Adjuvant	dose(μg/rabbit)	Adjuvant	dose(μg/rabbit)	Adjuvant	dose(μg/rabbit)
1st	FCA	350	ISA 201	350	ISA 206	350
2nd	FIA	350	ISA 201	350	ISA 206	350
3rd	None	350	None	350	None	350
4th	None	350	None	350	None	350

Note: FCA- Freund's complete adjuvant, FIA- Freund's incomplete adjuvant, ISA 201and ISA206 oil adjuvants were purchased from Seppic Company, France.

### Preparation of the colloidal gold-labeled 2C'3AB antigen

Colloidal gold was prepared according to the reported method[13]. 2C'3AB protein was diluted serially to give 10,20,30,40,50,60,70 and 80 μg/ml. 0.1 ml of each was added to colloidal gold solution (1 ml). Five minutes later, 10% NaCl (0.1 ml)was added each tube, mixed and allowed to sit for two tours. The optimum 2C'3AB protein concentration for labeling was the highest dilution color unchanged plus 20% protein. Protein was most efficiently absorbed onto the gold surface when its pI value was equal to pH value of colloidal gold. The pH value of colloidal gold was adjusted to 9.5, 9.0, 8.5, 8.0, 7.5, 7.0, 6.5 and 6.0 K2CO3 solution (0.1M). The optimum 2C'3AB protein was added to each and mixed for 10 minutes; 5%BSA was added to each to final concentration of 1% and mixed for 10 minutes. The optimum absorbing pH value was in maximum spectrum absorption value (*OD*_523 nm _). The optimum concentration of 2C'3AB antigen was added into the colloidal gold suspension and stirred for 30 minutes; 5% BSA was added to a final concentration of 1% and stirred for 20 minutes at room temperature; The colloidal gold-labeled antigen was centrifuged 2000 g at 4°C for ten minutes, and the precipitate discarded. The supernatant was centrifuged at 12000 g 4°C for sixty minutes. The supernatant was removed carefully and the precipitate washed twice with 0.01 M pH7.2 PBS buffer. The colloidal gold-labeled antigen was prepared when the volume was adjusted to its original volume with 0.01 M pH7.2 PBS buffer.

### Determination of the working concentrations of antigen and rabbit antibody

The colloidal gold-labeled 2C'3AB antigen was diluted serially as 3.0, 2.5, 2.0, 1.5, 1.0 and 0.5(O.D. value in *OD*_523 nm_), and sprayed them on glass fiber paper, which adhering on PVC board respectively to be primary strips to detect positive or negative reference samples. The working concentration for colloidal gold-labeled 2C'3AB antigen was based upon the time taken for the colour to develop and the intensity on the test line compared to the NC membrane background colour. To determine the working concentration of colloidal gold-labeled 2C'3AB antigen on glass fiber paper, 2C'3AB antigen was serially doubly diluted to 3.2 mg/ml,1.6 mg/ml,0.8 mg/ml and 0.4 mg/ml and blotted on test line of NC membrane, which adhering on PVC board respectively to be primary strips to detect positive or negative reference samples. The working concentration for 2C'3AB antigen on test line was determined depending on the color time, color depth on the test line and the background of NC membrane. To determine the working concentrations of colloidal gold-labeled 2C'3AB antigen on glass fiber paper and 2C'3AB antigen on test line of NC membrane, the rabbit 2C'3AB antibody was serially doubly diluted to 4.0 mg/ml, 2.0 mg/ml, 1.0 mg/ml and 0.5 mg/ml and blotted onto the control line of NC membrane, which adhering on PVC board respectively to be primary strips to detect positive reference samples. The working concentration of the 2C'3AB antibody on the control line was determined depending on the color situation on the test line and the control line.

### Preparation of the immunochromatographic strip, test procedure and test validation

**Figure 4:**
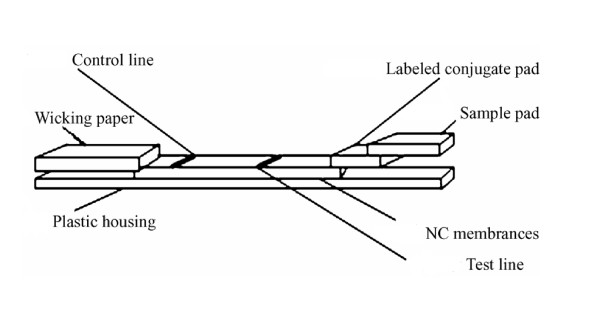
**The sketch map of gold-labeled immunochromatographic strip**. Wicking paper: The sample soaking pad, made of glass fiber. Labeled conjugate pad: Colloidal gold solution is coated on the pad. Sample pad: It was immersed into serum sample to soak.

The colloidal gold-labeled 2C'3AB antigen was dispensed onto the fiberglass (Millipore) at the speed of 15 μl/cm using a XYZ-3000 Dispensing Machine (Bio-Dot), dried and stored in a desiccator at room temperature. 2C'3AB antigen and rabbit antibody were dispensed onto the NC membrane (20 mm×300 mm) respectively according to work solution at the speed of 1.0 μl/cm to form the test line and control line 8 mm apart. The NC membrane was blocked and washed with 10% BSA in pH7.2 PBS (0.02 M), dried and stored in a desiccator at room temperature. The strip was assembled with sample pad, labeled conjugate pad, NC membrane, and absorbent pad on the PVC backing support board sequentially with a 1-2 mm overlap, and cut to 4.1 mm width strips with CM-4000 Cutter (Bio-Dot) (Figure 4). The sample pad of the strip was immersed into serum sample (the depth not more than marked immersion max line) for 30-60 seconds. When whole NC membrane was wetted, removed the strip and kept flat: the result became evident within 15 minutes. Test validation: when the clear red colors appear on both test line and control line, it is positive. When the clear red color appears only on control line, it is negative. When the clear red color appears on control line but a faint red color appears on test line, it is neutral. When the red color absents on control line, it is invalid.

### The validation test of the strip

The positive sera from cattle, swine and sheep provided by national foot-and-mouth disease reference laboratory of China were serially doubly diluted to 1:256 with pH7.2 PBS(0.02 M) respectively. The highest dilution was its sensitivity. The same procedure was repeated three times by different operators. The reference positive and negative sera provided by national foot-and-mouth disease reference laboratory of China were tested and judged. Thirty strips were taken randomly from three batches to test cattle, swine and sheep sera. To test the stability of the strips, the same batch of strips was stored at 37°C for one month or at 4°C for three months and tested the same reference samples, observing their stability. 387 serum samples were tested to evaluate the characteristics of the strip in contrast with existing commercial 3ABC antibody ELISA kit. The multi-vaccinated cattle sera were obtained as reference [14]described.

All animal work was approved by the Gansu provincial Administrative Committee for Laboratory Animals(Permission number: SYXK-GS-2010-0003).

## Results

### The expression, purification, identification and quantification of 2C'3AB recombinant protein

**Figure 5:**
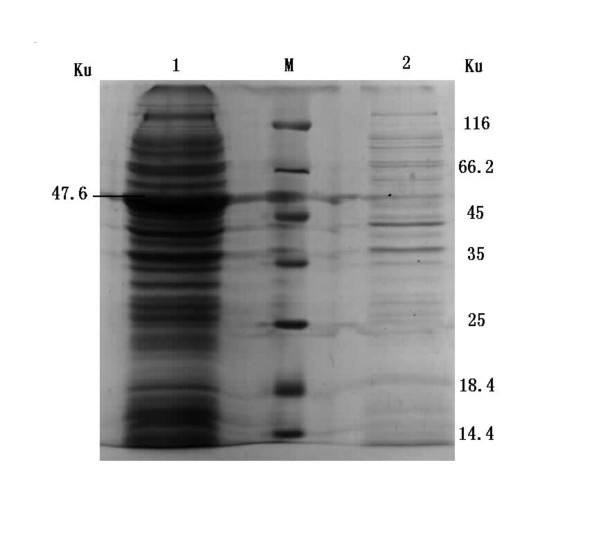
**Analysis of the expressed products by SDS-PAGE**. Lane M is protein molecular weight Marker; Line 1 shows recombinant protein after induced with IPTG.; Lane 2 shows recombinants before induced with IPTG.

**Figure 6:**
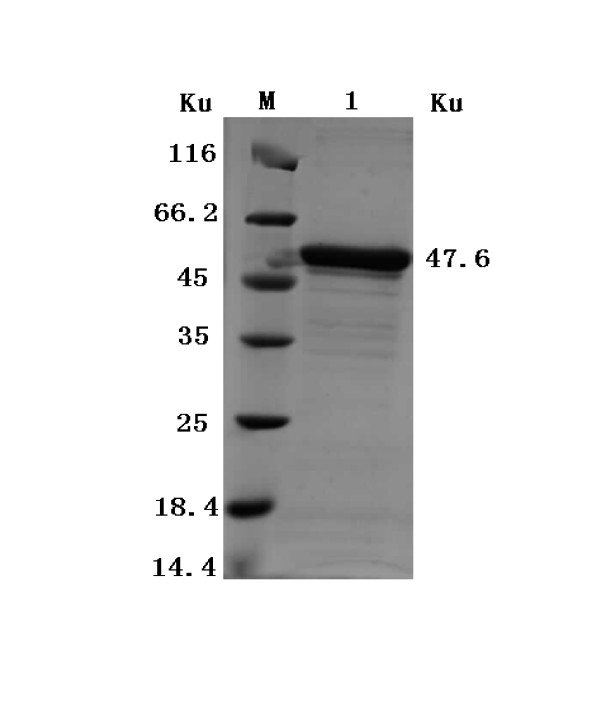
**Analysis of the purified recombinant proteins of 2C'3AB by SDS-PAGE**. Lane M is protein molecular weight Marker; Lane 1 is purified recombinant 2C'3AB protein

**Figure 7:**
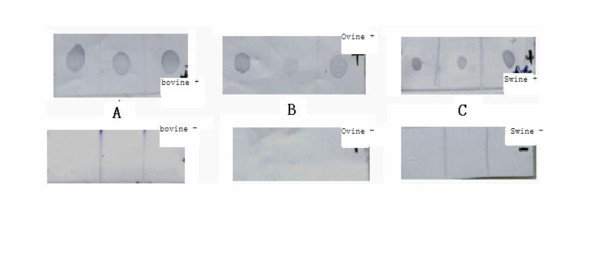
**The results of Dot-ELISA with antigen 2C'3AB**. A: Positive and negative serum of bovine; B: Positive and negative serum of ovine; C: Positive and negative serum of swine

**Figure 8:**
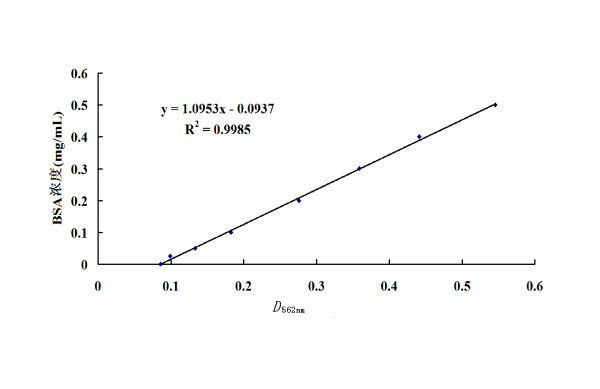
**Standard curve of the BCA**.

The 2C'3AB recombinant protein (predicted molecular mass = 47.6 kDa) was expressed (Figure 5). The purified 2C'3AB protein was obtained and analyzed (Figure 6), and its reactivity was determined with Dot-ELISA to test the positive and negative sera of cattle, pigs and sheep (Figure 7). The concentration of purified protein was 1.02 mg/ml (five times dilution, *OD*_562 nm _= 0.276) (Figure 8).

### Preparation and identification of 2C'3AB antibody

**Figure 9:**
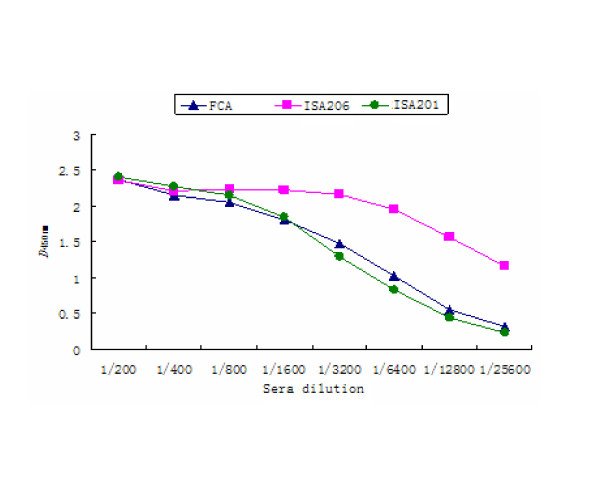
**The sera titers of immunized white rabbits with 2C'3AB antigen**.

**Figure 10:**
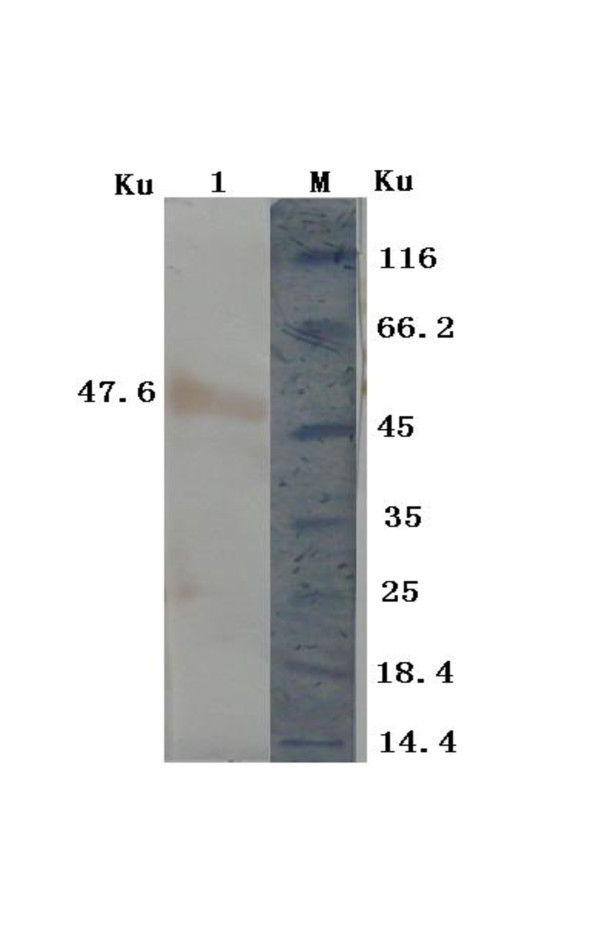
**Analysis of the purified 2C'3AB antibody with SDS-PAGE**. Lane M is protein molecular weight maker; Lanes 1 is purified antibody

**Figure 11:**
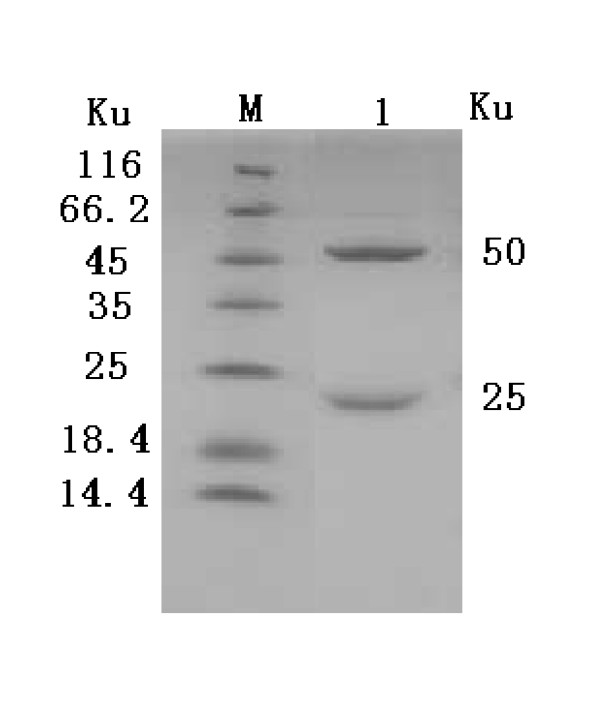
**Analysis of purified 2C'3AB antibody with Western blot**. Lane M is protein molecular weight Marker; Lane 1 is 2C'3AB antibody detected with Western blot.

**Figure 12:**
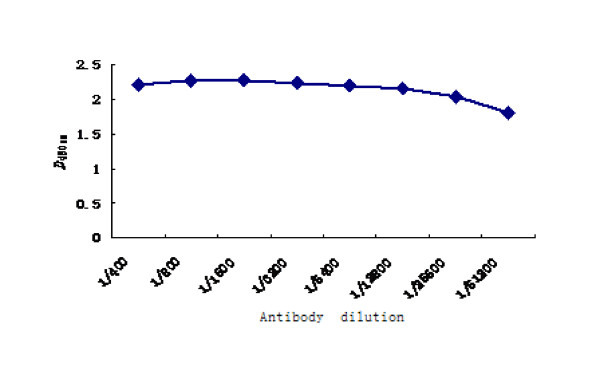
**2C'3AB antibody titer detection**.

The concentrations of the coated 2C'3AB antigen and the sheep-anti-rabbit IgG-HRP were determined with block titration method. The antigen was diluted by 1:200. The sheep-anti-rabbit IgG-HRP was diluted from 1:10000. The positive sera were from FMDV-challenged rabbits. The negative sera were from healthy rabbits. The optimal concentrations were obtained according to *OD*_450 nm_(Positive sample)/*OD*_450 nm_(negative sample)>2.1 and *OD*_450 nm_(negative sample) value was as low as possible(Table 2). The result showed that the optimal concentrations of coated antigen and the sheep-anti-rabbit IgG-HRP was 1:1600 (0.64 μg/ml) and 1:40000 respectively. The sera titers of vaccinated rabbits were tested with indirect ELISA. The antibodies were induced by 2C'3AB antigen emulsified with three different adjuvants. The sera titers induced by ISA 206 adjuvant were highest (Figure 9). Purified 2C'3AB antibody was analyzed with SDS-PAGE, showing a light chain (25 KDa) and a heavy chain(50 KDa)(Figure 10), and reacted to positive serum specifically with Western blot(Figure 11). Its titer was more than 1:51200 tested with indirect ELISA(Figure 12).

**Table 2:** The *OD*_450 nm _value of block titration

Dilution of sheep-anti-rabbit IgG-HRP	Rabbit sera	Antigen dilution 1:200	Antigen dilution 1:400	Antigen dilution 1:800	Antigen dilution 1:1600	Antigen dilution 1:3200	Blank control
1:10000	positive	3.374	3.279	3.202	2.665	2.010	0.021
	negative	0.076	0.087	0.065	0.076	0.055	0.033
1:20000	positive	2.827	2.691	2.444	2.174	1.891	0.032
	negative	0.066	0.067	0.065	0.045	0.076	0.053
1:40000	positive	2.525	2.398	1.891	**1.610**	1.472	0.034
	negative	0.062	0.054	0.042	**0.031**	0.030	0.042
1:80000	positive	1.667	1.407	0.990	0.632	0.415	0.035
	negative	0.045	0.034	0.031	0.029	0.026	0.010

### Preparation of colloidal gold particles

**Figure 13:**
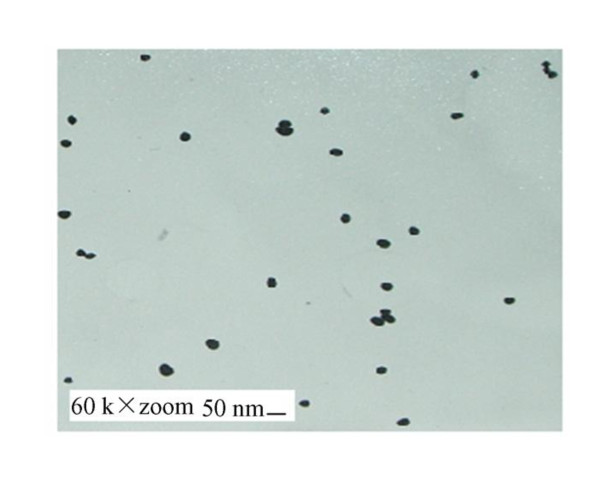
**TEM image of colloidal gold particles(× 60000)**.

The colloidal gold particles were homogeneous under electron microscope and their average diameter was 40.06 ±0.7 nm, which adapted to be labeled gold (Figure 13).

### The optimum conditions of the colloidal gold-labeled 2C'3AB antigen

**Figure 14:**
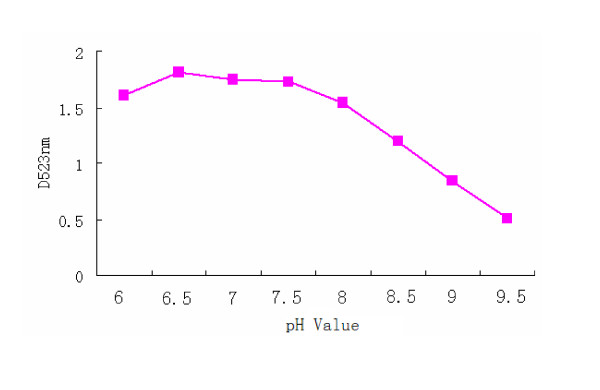
**The optimal pH of protein conjugated with colloidal gold**.

The optimal 2C'3AB protein concentration for labeling was 40 μg/ml. The optimal pH value of 2C'3AB protein conjugated with colloidal gold was 6.5(Figure 14).

### The working concentration for antigen and rabbit antibodies

The working concentration for colloidal gold-labeled 2C'3AB antigen was *OD*_523 nm _= 2.0. The working concentration for 2C'3AB antigen on the test line was 0.8 mg/ml. the working concentration for rabbit 2C'3AB antibody on control line was 1.0 mg/ml.

### The validation test of the strip

**Figure 15:**
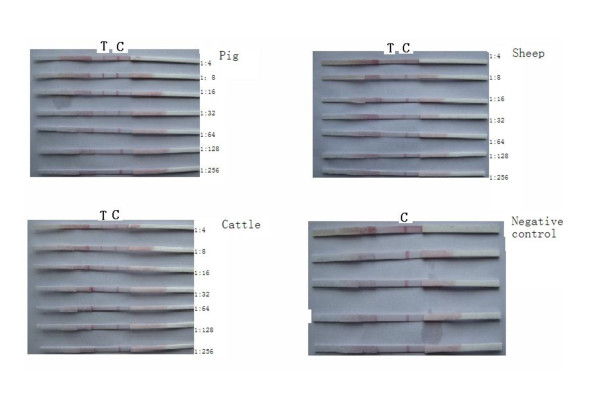
**Test results of sensitive assay of the strip**.

**Figure 16:**
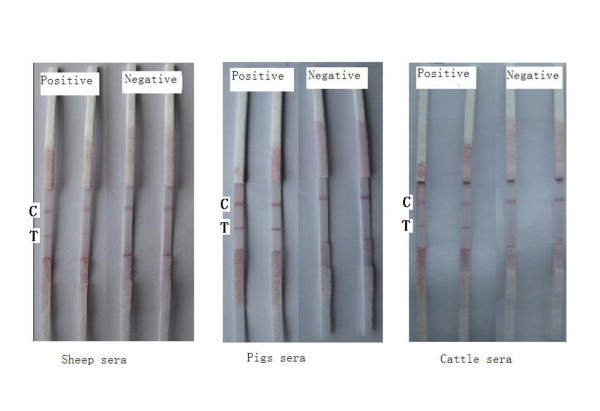
**Test results of specific assay of the strip**.

The colors on test lines were unclear when the dilutions of positive serum of pigs, cattle, and sheep were 1:64, 1:64, 1:32 respectively (Figure 15). A good specificity was validated assaying positive and negative sera of cattle, sheep and pigs (Figure 16). Thirty strips from three batches did not show color differences, validating their good repeatability.

The sensitivity and specificity of the strips did not changed obviously at 4°C in three months but the sensitivity reduced gradually at 37°C post 28 days. The high coincidence rate was obtained comparing the strips with existing commercial 3ABC antibody ELISA kit (Table 3). The coincidence rate of pigs negative serum, pigs vaccinated serum, pigs infected serum was 100%, 97.2%, 95.0%, respectively. The coincidence rate of cattle negative serum, cattle vaccinated serum, cattle infected serum was 100%, 96.7%, 98.0%, respectively. The coincidence rate of sheep negative serum, sheep infected serum was 97.6%, 96.3%, respectively.

**Table 3:** Comparison of the immunochromatographic strip and 3ABC ELISA kit

Sera amounts/Animals		The strip tests		3ABC ELISA tests		The coincidence		The coincident rate	
		Positive	Negative	Positive	Negative	Positive	Negative		
51/Negative pigs		None	51	None	51	None	51	100%	
36/Vaccinated pigs		1	35	None	36	None	35	97.2%	
20/Challenged pigs		7	13	8	12	5	12	95.0%	
36/Negative cattle		None	36	None	36	None	36	100%	
94/Vaccinated cattle	2^nd ^vaccination	1	11	1	11	1	11	100%	
	3^rd ^vaccination	1	9	1	9	1	9	100%	
	4^th ^vaccination	3	9	2	10	2	9	91.7%	
	5^th ^vaccination	4	8	4	8	4	8	100%	
	6^th ^vaccination	6	6	6	6	6	6	100%	96.7%
	7^th ^vaccination	7	5	6	6	6	5	91.7%	
	8^th ^vaccination)	7	5	6	6	6	5	91.7%	
	9^th ^vaccination	8	4	8	4	8	4	100%	
54/Challenged cattle		48	6	49	5	48	5	98%	
42/Negative sheep		1	41	None	42	None	41	97.6%	
54/Challenged sheep		29	25	27	27	27	25	96.3%	

## Discussion

The most important and effective routes of prevention and control of FMD are vaccination and elimination of infected animals in developing countries. The strategy currently utilized to kick off FMDV infected from vaccinated animals is to detect NSP antibodies. The immunochromatographic strip is a new technique including a cellulose membrane as the carrier and a colloidal gold labeled antigen or antibody as the tracer, which has some advantages over traditional immunoassays, such as procedure simplicity, rapid operation and immediate results, low cost, no requirements for skilled technicians or expensive equipment, so it is suitable for field assays of antibodies or antigens [15].

For FMD, an inactivated vaccine, generated from virus propagation in the cell line BHK-21, is commonly used. Although SPs and NSPs are expressed at the same time, some of the NSPs are eliminated during the process of vaccine production. However, dissociated NSPs and NSP complexes are difficult to eliminate and can induce antibodies after repeated vaccination, making it difficult to differentiate infected animals from vaccinated animals. A genetically engineered FMD vaccine is currently being developed and its biosafety is under investigation. However, in this case some NSP genes were altered to increase their expression and consequently NSP antibodies appeared after several rounds of vaccination. This also led to difficulties in differentiating infected animals from vaccinated animals. Therefore, it appears that regardless of the type of vaccine used, 3ABC antibodies can be induced after repeated vaccination, interfering with the diagnosis of infection or vaccination. Table 3 confirmed the phenomenon. Before third vaccination to twelve cattle, only one serum was positive, but eight sera were positive after ninth vaccination. FAO/OIE provides vaccine evaluation criteria to analyze NSP antibodies after three rounds of vaccination http://www.oie.int.

The reliability of detection of 3AB antibodies is higher than that of other NSPs[7]. Hence, ELISA methods to detect 3AB or 3A antibodies have been established in many laboratories around the world[16], and 3C protein was lower[8].

The FMDV-specific linear B-cell epitopes in 2C protein are mainly converged in the N-terminal[16]. A part of N-terminal portion of 2C protein gene and whole 3AB gene were connected to enrich B-cell epitopes and assay two sorts of antibodies of 2C and 3AB to improve the test accuracy. 2C antibodies can be detected in sera of infected cattle after one year, but it is absent in vaccinated cattle because 2C protein could be eliminated with cell debris during vaccine production due to it combining with cell members tightly, suggesting 2C protein is a reliable mark to distinguish infected from vaccinated animals[17]. Accordingly, detection 2C and 3AB antibodies simultaneously can improve the test accuracy.

Although seven FMDV serotypes exist around the world, the NSPs are highly conserved among serotypes, and detection of the NSPs antibodies has the additional advantage of serotype independence[18]. E. coli expression systems have several advantages such as short period, high efficiency, easy operation and a large amount of products, so we chose it to express recombinant 2C'3AB protein.

The specificity of a strip is the first consideration, because high probability of false positive or false negative one will obstruct its usage. Determination of optimal work concentrations of labeled 2C'3AB protein, 2C'3AB antigen on test line, and 2C'3AB antibody on control line are key factor to improve its specificity.

The validation tests need a long period of time and several different tests. In the present study, five tests of sensitivity, specificity, repeatability, stability and contrast were made, and good results were shown by testing 387 sera obtained from cattle, pigs and sheep. Thus, a new kind of strip applied in field simply and rapidly to differentiate infected from vaccinated animals was developed.

## Competing interests

The authors declare that they have no competing interests.

## Authors' contributions

ZXL was responsible for the research. XTL designed the research. LW and TJ carried out most of the experiments. ZJL designed part of experimets. DL wrote and revised the manuscript, PS, ZL, YMY, YFF, YMC carried out part of the experiment. All of the authors read and approved the final version of the manuscript.
